# *Tatupa grafei*, a new genus and species of Cylapinae (Heteroptera, Miridae) from Brunei Darussalam

**DOI:** 10.3897/zookeys.946.51780

**Published:** 2020-07-06

**Authors:** Veronica D. Tyts, Anna A. Namyatova, Claas Damken, Rodzay A. Wahab, Fedor V. Konstantinov

**Affiliations:** 1 Department of Entomology, Faculty of Biology, St. Petersburg State University, Universitetskaya nab. 7/9, St. Petersburg 199034, Russia; 2 All-Russian Institute of Plant Protection, Podbelskogo sh. 3, St. Petersburg 196608, Russia; 3 University of Tyumen, Volodarskogo ul. 6, Tyumen 625003, Russia; 4 Institute for Biodiversity and Environmental Research, Universiti Brunei Darussalam, Jalan Universiti, BE1410, Brunei Darussalam; 5 Zoological Institute, Russian Academy of Sciences, Universitetskaya nab. 1, St. Petersburg, 199034, Russian Federation

**Keywords:** Borneo, dipterocarp forest, morphology, *Rhinocylapus*-complex, taxonomy

## Abstract

A new genus and species, *Tatupa grafei* Tyts, Namyatova & Konstantinov, **gen. et sp. nov.** (Heteroptera, Miridae, Cylapinae, Fulviini), is described from Brunei Darussalam. A diagnosis, photographs of the dorsal habitus, scanning micrographs of selected morphological structures, and illustrations of male and female genitalia are provided for this new species. Its taxonomic placement within the subfamily Cylapinae is briefly discussed. A comparison with the morphologically most similar genus, *Proamblia* Bergroth, 1910, is made, and scanning micrographs of *Proamblia* are also provided.

## Introduction

Borneo is mostly covered with highly diverse tropical rainforests (e.g. Ashton 2010) and recognized as one of the biodiversity hotspots (e.g. Myers et al. 2000; de Bruyn et al. 2014). The major lowland forest formation of this island is dipterocarp forest, which is the most diverse ecosystem in the world (Davies and Becker 1996; Hédl et al. 2009). Borneo harbors a great number of arthropods, including numerous endemic species, many of them undescribed and at risk of extinction because of the intensive logging (Hédl et al. 2009; Ashton 2010; Berry et al. 2010; Giam et al. 2010). Brunei Darussalam is important for biodiversity conservation as it is least affected by the conversion of rainforests into palm oil plantations in comparison with Indonesia and Malaysia (Damken et al. 2017).

Although the area of Brunei Darussalam is relatively small, occupying only around 1% of Borneo, its insect fauna remains understudied. An important glimpse into the biodiversity of Brunei’s rainforests was provided by the canopy fogging study conducted by Nigel Stork in the early 1980s (Stork 1991); his study yielded more than 3000 insect species from just 10 trees. The construction in 1990 of the Kuala Belalong Field Studies Centre in the southern part of Temburong District, within the Batu Apoi Forest Reserve (later declared as Ulu Temburong National Park) provided the much needed permanent logistics to conduct fieldwork in a near-pristine lowland mixed dipterocarp forest and Kuala Belalong. The forest reserve has since become the type locality for many of the invertebrates recorded or described from the Sultanate (e.g. Damken et al. 2017; Gnezdilov 2015; Heiss 2011; Kočárek et al. 2017; Pfeifer et al. 2011; Ševčík et al. 2014; Tan et al. 2017; Wolski and Gorczyca 2012).

From 2013 to 2015, the third author conducted systematic field sampling of Heteroptera (Hemiptera) in various locations and forest types across the Sultanate. A regional (i.e. Borneo) reference collection was established for pristine forests for a group of tropical insects with both a moderate species diversity and moderate specimen abundance. The collection can be used to conduct future ecological studies, such as the impact of land-use change on tropical insect diversity. During this field survey, more than 400 species of Heteroptera were collected, including many hitherto undescribed species.

The hyperdiverse family Miridae, in the order Hemiptera, is well represented in Brunei, as most of its suprageneric groupings are most diverse in the tropics (e.g. Schuh and Slater 1995; Cassis and Schuh 2012). However, apart from the mirine *Kosmiomiris carvalhoi* Kim & Jung, 2019 (Kim et al. 2019), only taxa from the less species-rich subfamilies, Cylapinae (Gorczyca 1999, 2006; Wolski 2008; Wolski and Gorczyca 2006, 2007, 2012; Wolski et al. 2018) and Isometopinae (Akingbohungbe 2013; Taszakowski et al. 2020), have been recently recorded or described from Brunei Darussalam.

We describe here a new cylapine genus and species from the dipterocarp forest of Brunei Darussalam. Species of the subfamily Cylapinae live in litter or under bark, presumably are mycetophagous or some may be predacious, and are most abundant in subtropical and tropical forests (e.g. Gossner and Damken 2018; Namyatova et al. 2018; Namyatova and Cassis 2019; Wheeler 2001; Wolski and Gorczyca 2011; Yasunaga 2000; Yasunaga and Miyamoto 2006). This is one of the least diverse mirid subfamilies, as currently known, but many tropical taxa still await description.

## Material and methods

### Specimens

The holotype and six paratypes of the new species described in this paper will be deposited in the Universiti Brunei Darussalam Museum (UBDM), but are currently retained on loan in the private research collection of Claas Damken, Dunedin, New Zealand. Two paratypes of the new species are deposited in the Zoological Institute, Russian Academy of Sciences (ZISP). Each specimen was associated with a unique specimen identifier or USI (see Material examined section), and was entered into the Arthropod Easy Capture Specimen database (https://research.amnh.org/pbi/locality/). Additional information such as photographs of habitus and scanning electron micrographs of selected structures, georeferenced coordinates of each locality, specimens dissected and notes are accessible through the interface of the Heteroptera Species Pages (http://research.amnh.org/pbi/heteropteraspeciespage).

### Microscopy and illustrations

Observations, measurements, and digital dorsal color images were made with a Nikon SMZ 1500 stereomicroscope equipped with a Nikon D700 digital SLR camera. Drawings and images of the male and female genitalic structures were taken with a Leica DM2500 microscope equipped with a drawing attachment and a Leica DFC450 digital camera. Partially focused images of each specimen or structure were stacked using the HELICON FOCUS 7.5.4 software. Scanning electron micrographs of selected structures were taken using Tescan MIRA3 LMU, Quanta 3D DualBeam and Hitachi TM 3000 scanning microscopes. Specimens were uncoated, except Figures 2D, F, J, where legs were covered with 28 nm gold using a Leica EM SCD500 high vacuum film deposition system.

### Dissections

The genitalia were macerated in 10% KOH solution prior to dissection, cleared in distilled water, and then transferred to glycerin jelly for proper orientation. The aedeagus is described in repose.

### Terminology

The terminology used for male genitalia follows Konstantinov (2003), and, for females, follows Davis (1955).

### Measurements

The measurements were completed using a graticule and 10× eyepiece. All measurements are in millimeters (Table 1). Scale bars for genitalia equal 0.1 mm, scale bar for habitus equals 0.5 mm.

**Table 1.** Measurements (mm). Abbreviations: Cun–Clyp – distance between apex of clypeus and apex of cuneus in dorsal view, Head Length – distance between apex of clypeus and the highest point of vertex, AntSeg1 and AntSeg2 – length of antennal segments I and II, InterOcDi – width of vertex between inner margins of eyes in dorsal view.

		Length									Width				
		Body	Cun–Clyp	Pronotum	Head		AntSeg1		AntSeg2	Scutellum	Head	Pronotum	InterOcDi	Scutellum	Hemelytron
♂	Mean	4.58	3.94	0.73	0.84	0,87		1.72		0,52	1,04	1,28	0,49	0,60	1,45
	SD	0,14	0,15	0,05	0,06	0,05		0,07		0,03	0,03	0,10	0,01	0,03	0,08
	Range	0,27	0,35	0,13	0,13	0,13		0,23		0,08	0,08	0,25	0,03	0,08	0,20
	Min	4,45	3,75	0,65	0,78	0,80		1,60		0,48	1,00	1,13	0,48	0,55	1,35
	Max	4,73	4,10	0,78	0,90	0,93		1,83		0,55	1,08	1,38	0,50	0,63	1,55
	N	4	4	5	4	6		6		5	6	5	5	5	5
♀	Mean	4,98	4,35	0,80	0,95	0,94		1,82		0,59	1,12	1,44	0,55	0,71	1,71
	Min	4,93	4,33	0,80	0,93	0,90		1,70		0,55	1,10	1,43	0,55	0,70	1,65
	Max	5,03	4,38	0,80	0,98	0,98		1,95		0,63	1,13	1,45	0,55	0,73	1,83
	N	3	2	2	2	3		3		2	3	2	2	2	3

## Taxonomy

### Tatupa

Taxon classificationAnimaliaHemipteraMiridae

Tyts, Namyatova & Konstantinov gen. nov.

1C4DF2F6-7E7E-54FC-9B3D-BF0DD25853D1

http://zoobank.org/E7C59542-A3C8-4948-8D5C-2D1A72E75F5F

#### Type species.

*Tatupa grafei* Tyts, Namyatova & Konstantinov, sp. nov.

#### Diagnosis.

**Figure 1.**
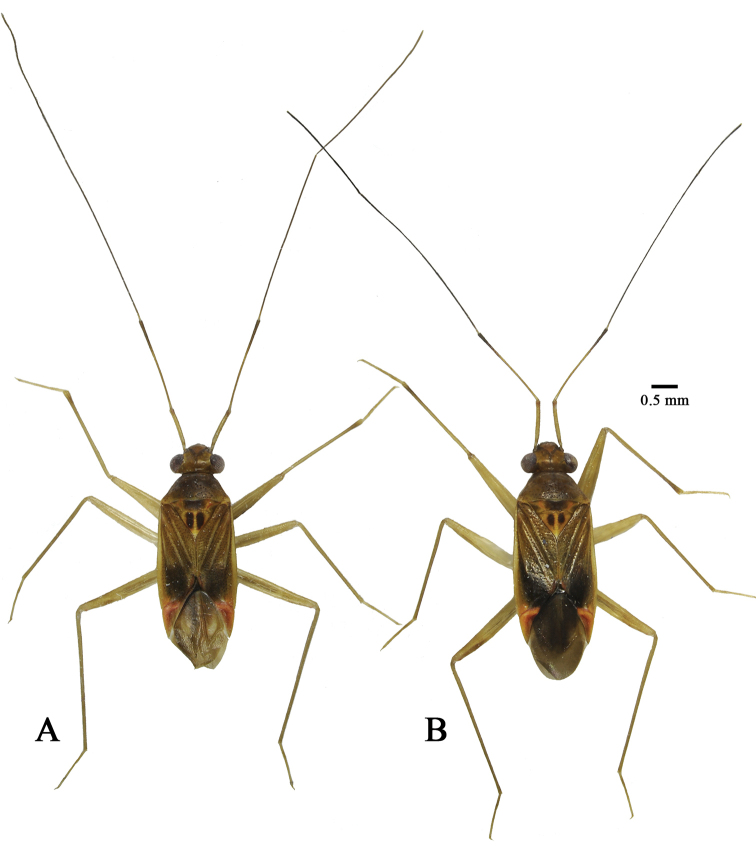
Habitus, dorsal view, *Tatupa grafei*. **A** male AMNH_PBI 00342925 **B** female AMNH_PBI 00342928.

**Figure 2.**
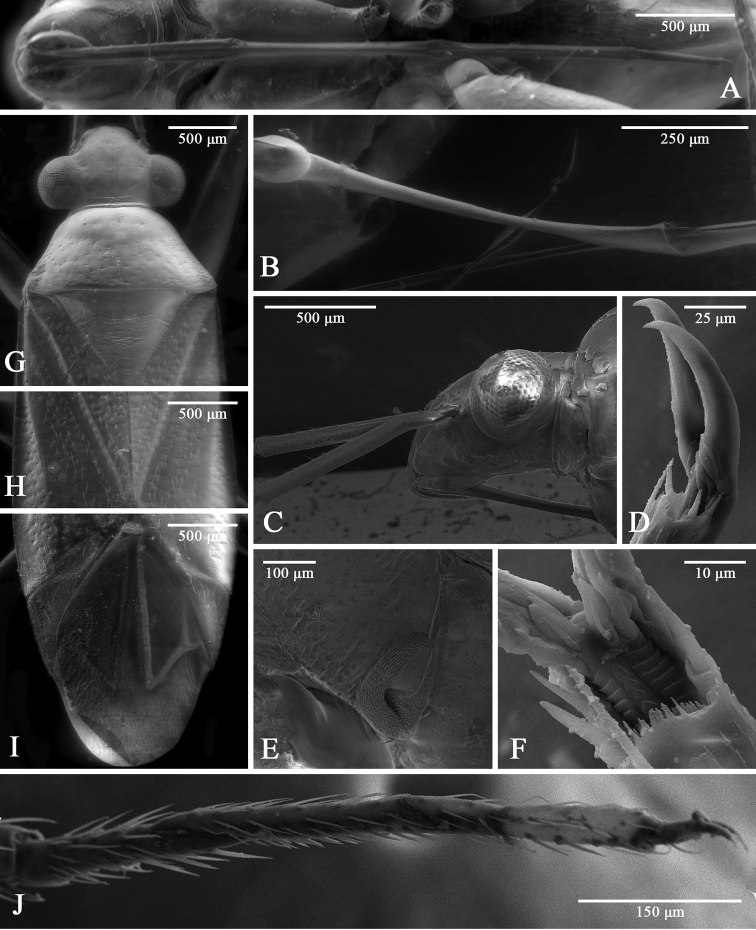
SEM images of *Tatupa grafei*. **A** labium and ventral view, female AMNH_PBI 00342926 **B** labial II segment, male AMNH_PBI 00342929 **C** head, lateral view, female AMNH_PBI 00343423 **D** hind pretarsus, lateral view **E** thoracic pleura, female AMNH_PBI 00342926 **F** hind pretarsus, ventral view, parempodia shown **G** head, pronotum, and scutellum, dorsal view, female AMNH_PBI 00342928 **H** clavus and corium, dorsal view, female AMNH_PBI 00342928 **I** corium, cuneus, and membrane, dorsal view, female AMNH_PBI 00342928 **J** fore tarsus.

**Figure 3.**
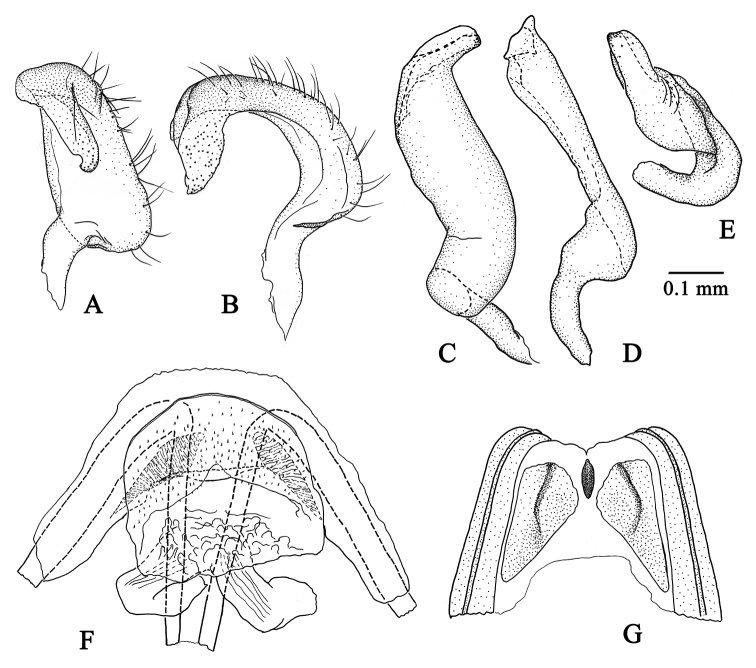
Male and female genitalia of *Tatupa grafei*. **A** left paramere, lateral view, AMNH_PBI 00342929 **B** left paramere, ventral view, AMNH_PBI 00342929 **C** right paramere, lateral view, AMNH_PBI 00342925 **D** right paramere, dorsal view, AMNH_PBI 00342925 **E** right paramere, caudal view, AMNH_PBI 00342925 **F** dorsal labiate plate, AMNH_PBI 00342926 **G** posterior wall of bursa copulatrix, AMNH_PBI 00342928.

**Figure 4.**
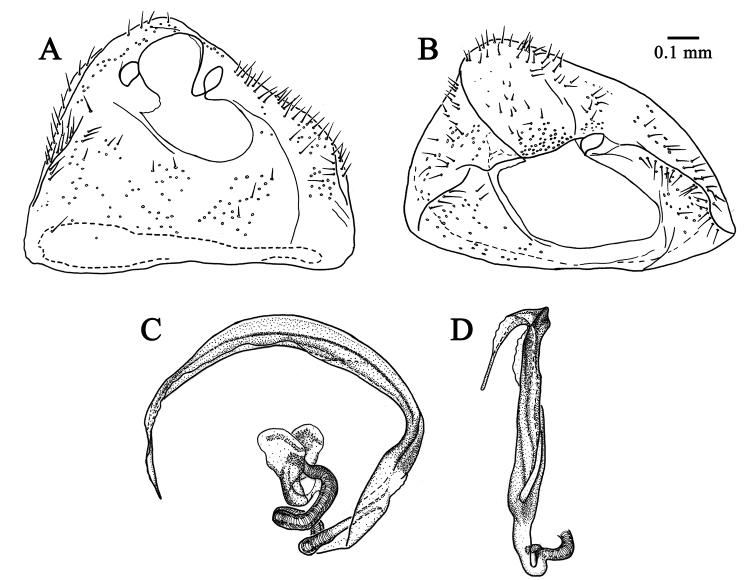
Male genitalia of *Tatupa grafei*. **A** genital capsule, dorsal view, AMNH_PBI 00342925 **B** genital capsule, caudal view, AMNH_PBI 00342925 **C** vesica, lateral view, AMNH_PBI 00342925 **D** vesica, ventral view, AMNH_PBI 00342924.

The new genus is recognized by the following combination of characters: head yellow to brownish yellow, sometimes with slightly darkened clypeus and usually with V-shaped dark marking on frons running from antennal fossa to midline (Fig. 1); head short in dorsal view (Fig. 2G), with ventrally directed apex of clypeus; portion of head anterior to eyes equal to eye length (Fig. 2C); labial segment I and II not subdivided (Fig. 2A–C); labrum without spines in both sexes; antennal fossa not adjoining to eye, separated from inferior eye margin by distance less than antennal fossa diameter and located at distance subequal to one-third of eye height from ventral margin of eye (Fig. 2C); antenna twice as long as body; antennal segment I distinctly longer than head width; pronotum entirely brownish yellow with slightly paler posterior angles (Fig. 1) and more sparsely punctured than hemelytron (Fig. 2G); calli weakly delimited and only slightly raised, occupying about half of pronotum, confluent at midline (Fig. 2G); pleura with round shallow punctures; peritreme of scent gland evaporative area twice as long as wide (Fig. 2E); scutellum flattened, not convex; aedeagus thin, C-shaped; vesica obvolute, with strongly sclerotized basal part and less sclerotized apically (Fig. 4C, D); posterior wall of bursa copulatrix with large, roughly triangular, keeled interramal sclerites (Fig. 3G).

#### Description.

**Male. *Coloration*.** (Fig. 1A) Head yellow to brownish yellow, sometimes with darkened, pale-brown to brown clypeus; frons usually with V-shaped dark marking running from antennal fossa to midline. Pronotum brownish yellow with slightly paler posterior angles.

***Surface and vestiture.*** Dorsum with whitish, scarce, short, adpressed simple setae (Fig. 2G–I); appendages and abdomen with similar but longer setae; antennal segment I covered with sparse, decumbent setae (Fig. 2C); segment II with dense adpressed setae on apical third and very sparse setae basally; pleura without setae (Fig. 2G). Dorsum moderately shiny; vertex distinctly shiny (Fig. 1); mesopleuron slightly rugose (Fig. 2E); scutellum rugose (Fig. 2G); evaporative scent gland area and scutellum matt; posterior part of pronotum, mesopleuron, clavus, and corium with distinct deep often pale punctures, some specimens with darkened punctures on hemelytron; pronotum more sparsely punctured than hemelytron; head, anterior part of pronotum, propleuron, scutellum and abdomen with round shallow punctures (Fig. 2E, G–I).

***Structure*.** Body elongate, more than three times as long as width across hemelytron.

***Head.*** Sloping, wider than long in dorsal view (Fig. 2C), short as seen from above; eye contiguous with pronotum; vertex wider than eye diameter (Fig. 2G); in lateral view head distinctly longer than high; portion of head anterior to eyes equals to eye diameter; clypeus moderately extending forward; apical part of clypeus directed ventrally; eyes relatively large, occupying slightly less than two-thirds of head height; antennal fossa removed from eye at distance less than antennal fossa diameter; distance between antennal fossa and ventral margin of eye subequal to one-third of eye height; buccula slightly shorter than distance between pronotum and buccula, gradually diminishing posteriorly and reaching just behind antennal fossa (Fig. 2C); labium thin and long, surpassing abdominal segment VIII and nearly reaching apex of abdomen; segments I and II not subdivided (Fig. 2A, C); segment I surpassing posterior margin of head, reaching or almost reaching forecoxae; segments I, II, and III subequal in length, each of them twice as long as segment IV (Fig. 2A–C); antenna twice as long as body; segment I and II cylindrical; segment I slightly incrassate towards apex, subequal to half of segment II; segments III and IV filiform (Fig. 1A).

***Thorax.*** Collar narrow, delimited with shallow suture laterally, suture distinct dorsally; lateral margins of pronotum slightly carinate on basal part; posterior margin bisinuate; calli mostly fused and slightly raised, occupying slightly less than half of pronotum, confluent at midline, with shallow furrow; mesoscutum exposed, with ridges laterally; scutellum with acute apex, flattened (Fig. 2G); mesepimeral apodeme arcuate, slit-like; metathoracic spiracle slit-like narrow, not surrounded with microsculpture; metathoracic scent gland evaporative area oval; peritreme twice as long as wide, flattened (Fig. 2E); metepimeron narrow.

***Hemelytron.*** Claval commissure 1.5 times as long as scutellum; clavus with distinct projecting claval vein, forming ridge; medial fracture distinct, surpassing middle of corium; R+M distinct; embolium clearly delimited only on basal half; cuneal fracture not incised (Fig. 2G–I). Membrane with two cells; outer cell surpassing apex of cuneus, longer than half of membrane, with acute angle; inner cell small, near middle of cuneus (Fig. 2I).

***Legs.*** Coxae slightly elongate; forecoxa longer than others; hind coxa wider than others; femora narrow; forefemur wider than hind and middle femora; hind femur longer than others; tarsus three-segmented; length of segments of hind tarsus subequal (Fig. 2J); pretarsus with three rows of lamellae on unguitractor close to each other and with acute lamellae on medial row (Fig. 2F); claws slightly curved, without tooth apically (Fig. 2D).

***Genitalia.*** Genital capsule distinctly wider than long, apically asymmetric, with shallow longitudinal sutures at sides, clothed with almost evenly distributed short setae; ventral wall of genital capsule distinctly longer than dorsal, hoodlike in posterior view (Fig. 4A, B); aperture of genital capsule wide, without supragenital bridge; right paramere oblong, with long scarce setae on dorsal side, basally curved, apically beakshaped, flattened and covered with minute denticles (Fig. 3C–E); left paramere slightly wider but shorter than right paramere, hook-shaped, covered with long erect setae on dorsal side, basally widened, apically tapering, with small excision and minute denticles at apex (Fig. 3A, B); aedeagus thin, C-shaped, phallotheca moderately sclerotized, narrow, tightly adjoining to vesica along entire length; vesica C-shaped, long and narrow, obvolute, apically tapering, with strongly sclerotized basal part and somewhat weaker sclerotized apically; basal part of ductus seminis running from phallobase to base of vesica equipped with sclerotized ribs; apical part of ductus seminis inside vesica membranous, hardly visible; secondary gonopore subapical, indistinct, without any sculpturing (Fig. 4C, D).

**Female. *Coloration*.** As in male, generally darker.

***Surface and vestiture*.** As in male.

***Genitalia.*** Dorsal labiate plate entirely membranous, very thin, covered with tiny spinules, lateral oviducts thick (Fig. 3F); posterior wall of bursa copulatrix with large, roughly triangular, keeled interramal sclerites at sides and small elongate sclerite located on midline (Fig. 3G); vestibulum membranous, without sclerites encircling vulva; ventral wall membranous, without sclerotizations.

#### Etymology.

The name of the new genus is a random combination of letters. The gender is feminine.

#### Remarks.

Morphological examination of the new genus indicates that it belongs to the *Rhinocylapus*-complex of the tribe Fulviini sensu Namyatova and Cassis (2019). It has been demonstrated, using molecular and morphological data, that the *Rhinocylapus*-complex is a distinct group, differing from other representatives of the Cylapinae in the structure of the pleura, shape of the parameres, and sclerotization of the posterior wall of the bursa copulatrix (Namyatova and Cassis 2019). In particular, the *Rhinocylapus*-complex has reduced metathoracic evaporative area not reaching the base of the hind coxa (Fig. 2G) with a flattened peritreme and the metathoracic spiracle without microsculpturing. The right paramere in this group is almost straight, widened medially, and with a cone-shaped outgrowth subapically. The left paramere is hook-like with the apical part elongate dorsally, and the posterior wall of the bursa copulatrix has two large symmetrical sclerites (see Namyatova and Cassis 2019 for details). *Tatupa* possesses all the diagnostic characters of the *Rhinocylapus*-complex.

The new genus differs from other genera of the *Rhinocylapus*-complex by the characters given in the diagnosis. It differs from most of the genera of this group in the shape of the head, which is declivous in males and females (Fig. 2C) and short as seen from above (Fig. 2G), and in having the labrum without spines. *Tatupa* also is unique within this group in having a C-shaped aedeagus with a strongly sclerotized obvolute vesica that is even more sclerotized basally and without any separate sclerites (Fig. 4C, D). A similar head is present in *Mycetocylapus* (Namyatova and Cassis 2019: figs 6F, 9A, D), but *Tatupa* differs from this genus in having calli that are far less raised and occupy only about a half of pronotum (Namyatova and Cassis 2019: compare fig. 2G and figs 6A, B, 9B, E), and an oval and more elongate peritreme (Fig. 2E); in *Mycetocylapus* a peritreme is small and rounded (Namyatova and Cassis 2019: fig. 6R). Additionally, the vesica in *Mycetocylapus* is membranous and only slightly sclerotized at its base (Namyatova and Cassis 2019: fig. 7A–C, H).

**Figure 5.**
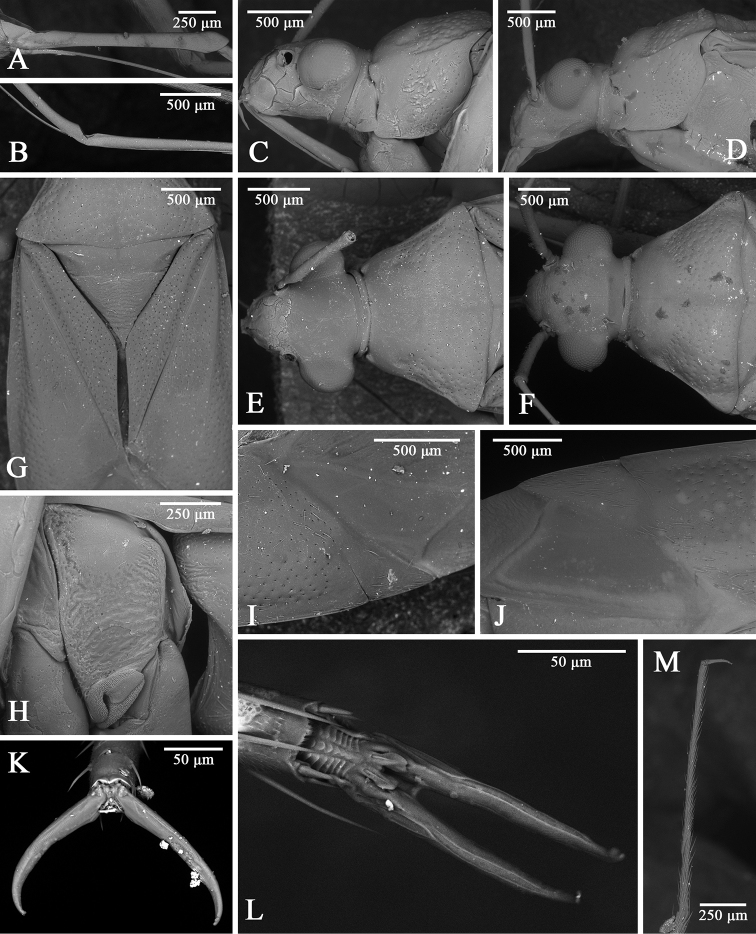
SEM images of *Proamblia* sp. **A** labial I segment, female UNSW_ENT 00045352 **B** labial II segment, female UNSW_ENT 00045352 **C, D** head and pronotum, lateral view, female UNSW_ENT 00045447, female UNSW_ENT 00045352 **E, F** head and pronotum, dorsal view, female UNSW_ENT 00045447, female UNSW_ENT 00045352 **G** scutellum, clavus, and corium, dorsal view, female UNSW_ENT 00045447 **H** thoracic pleura, female UNSW_ENT 00045447 **I, J** cuneus and membrane, dorsal view, female UNSW_ENT 00045447, female UNSW_ENT 00045352 **K** pretarsus, dorsal view, male UNSW_ENT 00045339 **L** pretarsus, ventral view, parempodia shown, male UNSW_ENT 00045339 **M** hind tarsus, male UNSW_ENT 00045351.

*Tatupa* presumably is most closely related to *Proamblia*, as these two genera cannot be differentiated from each other in head shape, as well as body size and proportions. Both genera also have similar punctation, and in particular, the posterior part of pronotum, mesopleuron, clavus, and corium are covered with distinct deep punctures, whereas the anterior part of pronotum, propleuron, scutellum, and abdomen have round shallow punctures (Figs 2G–I, 5C–J). *Tatupa* differs from *Proamblia* in the flattened, not convex scutellum and pronotum that is more sparsely punctured than the hemelytron (Fig. 2G), whereas in *Proamblia* the scutellum is distinctly convex and the pronotum usually is as densely punctured as the hemelytron, with punctures sometimes dense only along posterior margin of calli (Fig. 5E–G). Moreover, *Tatupa* clearly differs from *Proamblia* in the structure of strongly sclerotized, C-shaped aedeagus (see above) (Fig. 4C, D), whereas in *Proamblia* the aedeagus is slightly curved, elongate, distinctly membranous, and the vesica has single endosomal sclerite and a relatively long sclerotized portion of ductus seminis (Wolski 2010: fig. 8A, B, G, F). The new genus also has almost triangular sclerites on the posterior wall of bursa copulatrix, each having a ridge (Fig. 3G), whereas in *Proamblia* those sclerites are more elongate and without ridges (Namyatova and Cassis 2019: fig. 15C).

In most cases, *Tatupa* and *Proamblia* can be differentiated using color pattern. The new genus possesses a yellow to brownish-yellow head, sometimes with a slightly darkened clypeus and with a V-shaped dark marking running from the antennal fossa to the midline of the frons (Fig. 1), whereas head color in *Proamblia* varies from brownish to almost entirely dark brown, sometimes with paler areas on the gula, the vertex or near the eye margin, and without a V-shaped dark marking on the frons. Additionally, *Tatupa* has a more or less uniform brownish-yellow pronotum with slightly paler posterior angles (Fig. 1), whereas the pronotum in *Proamblia* varies from brownish to dark brown and typically has a yellow posterior margin and/or yellow stripes on the pronotum and/or yellow markings on the calli.

### Tatupa grafei

Taxon classificationAnimaliaHemipteraMiridae

Tyts, Namyatova & Konstantinov sp. nov.

43E7F77C-35AB-5E83-929F-E462E89B28BC

http://zoobank.org/C571F541-BFE1-4816-A36C-E7BB301A6B4A

#### Material examined.

***Holotype.*** Brunei Darussalam • 1♂; Temburong District, Temburong National Park; 4.5178N, 115.1778E; 13 Nov. 2013; C. Damken leg.; mixed dipterocarp forest, bark with fungi, dead tree; DBH 110 cm; 2000–2200 hours; AMNH_PBI 00342925, belalong.02143; UBDM.

***Paratypes.*** Brunei Darussalam • 3 ♀; same data as for holotype; AMNH_PBI 00342928, belalong.02135; ZISP; AMNH_PBI 00342926, belalong.02261; AMNH_PBI 00343423, belalong.02144; UBDM • 2 ♂; same data as for holotype; AMNH_PBI 00342927, belalong.02263; AMNH_PBI 00342924, belalong.02262; UBDM • 3 ♂; Temburong District, Temburong National Park, Ashton trail; 4.5333N, 115.15E; 15 Jan. 2014; C. Damken leg.; mixed dipterocarp forest, under bracket fungi, hand collected; 2000–2200 hours; AMNH_PBI 00342929, belalong.02264; UBDM; AMNH_PBI 00342930, belalong.02265; ZISP.

#### Diagnosis.

As in generic diagnosis.

#### Description.

**Male. *Coloration*** (Fig. 1A). Head yellow to brownish yellow, sometimes with darkened, pale-brown to brown clypeus; frons usually with V-shaped dark marking running from antennal fossa to midline; eye dark brown with reddish tinge; labium yellow, with mostly dark-brown segment IV; antennal segment I dirty yellow, with darkened base and reddish or brownish tinge apically; segment II yellow, gradually darkened to dark brown towards apex; segment III brown to dark brown with pale yellow base; segment IV brown to dark brown.

***Thorax.*** Pronotum brownish yellow with slightly paler posterior angles; exposed part of mesonotum yellow, somewhat darkened at middle, usually with reddish tinge at sides; scutellum yellow with paired brown longitudinal markings, sometimes with reddish tinge near anterior angles; thoracic pleura brownish yellow, sometimes with red tinge; hemelytron yellow to pale brown; clavus with whitish stripe along claval vein; corium with whitish stripes along medial fracture and R+M vein, and darker pale brown to brown large marking medioapically; embolium whitish; cuneus yellow with reddish tinge, sometimes indistinct; membrane brown, larger cell sometimes pale brown apically.

***Legs.*** Coxae whitish; femora, tibiae, and tarsi yellow to pale brown.

***Abdomen.*** Yellow with reddish tinge, brown laterally.

***Structure and vestiture.*** As in generic description.

***Ratios*.** Body 3.0–3.3× as long as wide, 3.4–4.0× as long as pronotum width, head 1.2–1.3× as wide as long, vertex 1.7–1.9× as wide as eye, antennal segment I. 1.7–1.9× as long as vertex, segment II 1.9–2.1× as long as segment I, 3.4–3.7× as long as vertex, 1.6–1.7× as long as head width, 1.3–1.4× as long as pronotum base width; pronotum 1.7–1.8× as wide as long, 1.1–1.3× as wide as head, scutellum 0.8–0.9× as long as wide.

**Female. *Coloration*.** As in male, generally darker.

***Surface and vestiture.*** As in generic description.

***Ratios*.** Similar to male, but body generally larger and head longer in lateral view. Body 2.7–3.0× as long as wide, 3.4–3.5× as long as pronotum width, head 1.2× as wide as long, vertex 1.9× as wide as eye, antennal segment I 1.7–1.8× as long as vertex, segment II 1.8–2.2× as long as segment I, 3.1–3.3× as long as vertex, 1.5–1.8× as long as head width, 1.2× as long as pronotum base width, pronotum 1.8× as wide as long, 1.3× as wide as head, scutellum 0.8–0.9× as long as wide.

#### Distribution.

Known only from the type locality, Brunei Darussalam, Temburong National Park.

#### Natural history.

Collected from dipterocarp forest, under bracket fungi and bark with fungi from dead trees.

#### Etymology.

The new species is named after Professor Ulmar Grafe of the Universiti Brunei Darussalam for his generous help and advice during the field work of the third author and for his continuous and invaluable support of ecological research in Brunei Darussalam in general.

## Supplementary Material

XML Treatment for Tatupa

XML Treatment for Tatupa grafei
